# The Association between Educational Level and Cardiovascular and Cerebrovascular Diseases within the EPICOR Study: New Evidence for an Old Inequality Problem

**DOI:** 10.1371/journal.pone.0164130

**Published:** 2016-10-06

**Authors:** Fulvio Ricceri, Carlotta Sacerdote, Maria Teresa Giraudo, Francesca Fasanelli, Giulia Lenzo, Matteo Galli, Sabina Sieri, Valeria Pala, Giovanna Masala, Benedetta Bendinelli, Rosario Tumino, Graziella Frasca, Paolo Chiodini, Amalia Mattiello, Salvatore Panico

**Affiliations:** 1 Unit of Epidemiology, Regional Health Service, Grugliasco, Turin, Italy; 2 Unit of Cancer Epidemiology, University of Turin and Città della Salute e della Scienza Hospital, Turin, Italy; 3 Department of Mathematics, University of Turin, Turin, Italy; 4 Epidemiology and Prevention Unit, Fondazione IRCCS Istituto Nazionale dei Tumori, Milan, Italy; 5 Molecular and Nutritional Epidemiology Unit, Cancer Research and Prevention Institute-ISPO, Florence, Italy; 6 Cancer Registry, Department of Prevention, ASP, Ragusa, Italy; 7 Department of Physical and Mental Health and Prevention, Second University of Naples, Naples, Italy; 8 Department of Clinical and Experimental Medicine, University of Naples Federico II, Naples, Italy; University of Bologna, ITALY

## Abstract

**Background:**

A consistent association has been reported between low socioeconomic status (SES) and cardiovascular events (CE), whereas the association between SES and cerebrovascular events (CBVD) is less clear. The aim of this study was to investigate the association between SES (measured using education) and CE/CBVD in a cohort study, as well as to investigate lifestyle and clinical risk factors, to help to clarify the mechanisms by which SES influences CE/CBVD.

**Material and Methods:**

We searched for diagnoses of CE and CBVD in the clinical records of 47,749 members of the EPICOR cohort (average follow-up time: 11 years). SES was determined by the relative index of inequality (RII).

**Results:**

A total of 1,156 CE and 468 CBVD were found in the clinical records. An increased risk of CE was observed in the crude Cox model for the third tertile of RII compared to the first tertile (hazard ratio [HR] = 1.39; 95% confidence interval [CI] 1.21–1.61). The increased risk persisted after adjustment for lifestyle risk factors (HR = 1.19; 95%CI 1.02–1.38), clinical risk factors (HR = 1.35; 95%CI 1.17–1.56), and after full adjustment (HR = 1.17; 95%CI 1.01–1.37). Structural equation model showed that lifestyle rather than clinical risk factors are involved in the mechanisms by which education influences CE. No significant association was found between education and CBVD. A strong relationship was observed between education and diabetes at baseline.

**Conclusion:**

The most important burden of inequality in CE incidence in Italy is due to lifestyle risk factors.

## Introduction

Many epidemiological studies have shown that low socioeconomic status (SES) is related to an increased risk of several chronic diseases, such as tumors [1], respiratory diseases [2], diabetes [3,4], as well as cardiovascular events (CE), and cerebrovascular events (CBVD) [5,6].

Over the last 30 years, almost all the studies on the topic from Western Europe and the USA have agreed that people with lower SES are more prone to experience and die from CE or CBVD [7–12]. A recent systematic review pointed out a dose-effect social gradient in the incidence and mortality of CE [13], strengthening the causal relationship of this association. Regardless of the indicator of SES used (educational level, social position, income, etc.), the association between SES and CE and CBVD has been generally consistent, although there have been considerable variations in the size of differences between low and high SES groups [14].

The major concern on this topic is that social inequalities in the incidence of and mortality from CE and CBVD have increased over time [15], making this a compelling public health problem even in Europe. SES does not have a direct biological effect on disease, and the biological pathway of the association between SES and CE or CBVD has only been partially explained in the literature. The clinical risk factors normally employed to explain this relationship (hypertension, hypercholesterolemia, and diabetes) account for between 25% and 80% of CE or CBVD in large epidemiological cohorts [16–18]. Thus not only do previous studies explain only a fraction of the effect of SES on CE/CBVD, but they also usually do not include risk factors in a hypothetical biological pathway.

It is possible that SES plays a causal role in some chronic diseases via related conditions like anxiety and depression, which might lead to more risky behaviors (i.e., smoking, alcohol consumption, and unhealthy diet) [19]. On the other hand, at least a part of the social gradient in chronic disease risk could be due to psychobiological factors, which stimulate the central nervous system through neuroendocrine and immune activation [20]. This mechanism could lead to an increase in clinical risk factors such as hypertension, hypercholesterolemia, and insulin resistance.

The aim of this study was to investigate the association between SES (measured using educational level), CE, and CBVD in a Mediterranean cohort of about 47,000 individuals using the relative index of inequality (RII), an index based on education that allows investigators to control for birth cohort, gender, and region of residence. The study also aimed to analyze the prevalence of lifestyle and clinical risk factors that potentially lie on the biological pathway of the association between education and CE and CBVD, in order to help in clarifying the mechanisms by which SES influences CE and CBVD.

## Material and Methods

### Study population

The EPICOR study consists of the Italian cohort of the European Prospective Investigation into Cancer and Nutrition (EPIC), but studies cardiovascular and cerebrovascular outcomes. From 1993 to 1998 a total of 47,749 participants (15,171 men and 32,578 women) were recruited into the EPIC study from five centers in Italy: Turin and Varese (North-West), Florence (Center), and Naples and Ragusa (South). Methods of recruitment have been described previously [21]. Briefly, all participants completed a detailed, validated questionnaires on dietary habits and lifestyle factors. The EPICOR study was approved by the ethics committee of the Human Genetics Foundation (Turin, Italy). The study complies with the Helsinki declaration, and all participants gave informed written consent to use clinical data for research.

### Assessment of CE, CBVE, and death

Participants with prevalent or incident CE (major coronary events, myocardial infarction) and CBVD (major cerebrovascular events, ischemic and hemorrhagic stroke) were identified through linkage to hospital discharge records and/or through direct evaluation of medical notes and/or questionnaire information. Vital status was evaluated through linkage with municipality registers. Suspected deaths from CE and CBVD were identified in mortality files. Death was categorized as CE/CBVD death after verification against hospital discharge and clinical records. A case was defined fatal if the participant died within 28 days of diagnosis. Clinical records were always retrieved to verify and confirm CE and CBVD, using MONICA criteria (MONICA manual could be found at: www.ktl.fi/publications/monica/manual/part1/i-1.htm). Details on case ascertainment were described in [22–23].

Participants were followed for the events of interest from study entry until death, emigration, or end of the follow-up period, whichever occurred first. The end of follow-up for CE and CBVD varied by center (from 2003 to 2008).

We excluded participants with prevalent CE and CBVD (i.e., occurring before study entry) as well as those with missing information on educational level, which resulted in a final study sample of 43,791 participants,

### Lifestyle and clinical risk factors

Information on lifestyle risk factors (smoking status, alcohol consumption, physical activity, body mass index (BMI), adherence to Mediterranean diet, and total energy intake) and clinical risk factors (hypertension, hypercholesterolemia, and type 2 diabetes) were taken from questionnaires.

### Calculation of relative indices of inequality

We used educational level as an indicator of SES. Information on educational level was taken from questionnaires and was categorized as: primary school or none (low educational level), vocational or other secondary school (middle educational level), and university or vocational postsecondary school (high educational level).

We then calculated RII in order to overcome the issue of possible differences in the proportion of subjects in the educational levels across regions, genders, and 10-years birth groups. The RII was attributed on the basis of the rank of participant distribution within strata for center, birth cohorts, and gender [24]. The midpoint of the cumulative proportional distributions of each educational level was used to compute the RII score. Details and examples of such computations can be found in Sacerdote et al [3]. The RII score was then divided into tertiles, where the first tertile represented the highest level of education.

### Statistical analysis

Distributions of exposures were presented in the overall cohort and in CE and CBVD using means and standard deviations for quantitative variables and absolute frequencies and percentages for qualitative variables. Univariate differences between participants with or without such events were tested using t-tests or chi-square tests, as appropriate. Moreover, the distributions of variables across tertiles of RII were presented and differences were tested using 1-way analysis of variance and chi-square tests, as appropriate. Hazard ratios (HRs) for all outcomes of CE (major CE, myocardial infarction) and CBVD (major CBVD, ischemic and hemorrhagic stroke) and tertiles of RII, using the first tertile as the reference category, were computed using semi-parametric Cox models. The proportional hazard assumptions were tested using scaled Schoenfeld residuals. Crude models were stratified by center only. Three multivariable models were also built: 1) adjusting for lifestyle risk factors: smoking status (current, former, and never), alcohol consumption (yes/no), physical activity (low, moderate, and high), Italian Mediterranean Index (continuous) [25], BMI (continuous), and total energy intake (continuous); 2) adjusting for clinical risk factors: baseline hypertension, hypercholesterolemia, and prevalent diabetes; 3) adjusting for all variables in the previous models. All models were built adjusted for age and sex and were stratified by center. A few number of subjects with some missing values for the mentioned variables were excluded from the analyses. We also performed a separated analysis by gender (for females we adjusted also for menopausal status in adjusted model 1 and 3). In order to understand the confounding effects of each adjustment variable, different models were built, adding each variable separately to the crude model.

Logistic regression models (both crude and adjusted for the aforementioned lifestyle risk factors) were performed to investigate the relationship between RII and 1) CE and CBVD and 2) clinical risk factors. All analyses were performed using STATA V.13.

### Structural Equation Model

A Structural Equation Model (SEM) [26] was built in order to help in clarify the contribution of lifestyle and clinical risk factors in the mechanisms by which SES influences CE [27]. We used SEMs because they allow to construct a system of equations in which a variable can be the outcome in one equation and the predictor in another. Moreover, a not measured variable, called latent variable, can be introduced into the model if there are variables that are able to explain it [28].

**Fig 1:**
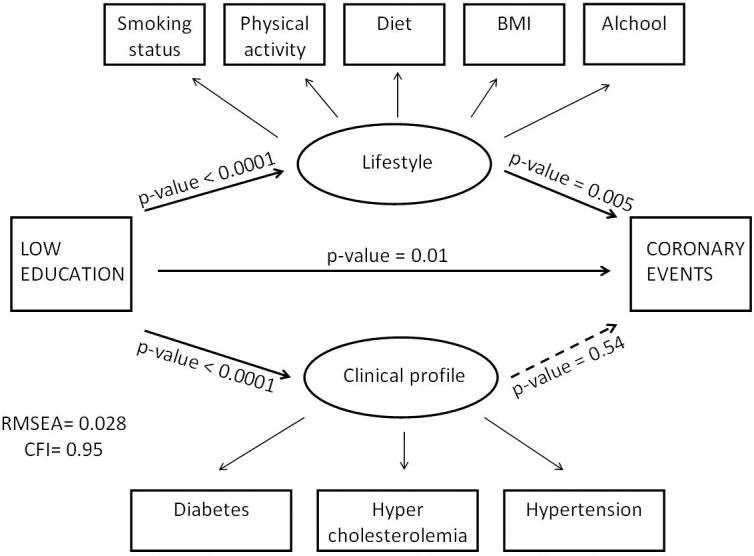
Direct Acyclic Graph (DAG) for the association of low educational status and Major Cardiovascular Events with results from Structural Equation Model.

In the model we drawn (Fig 1), we supposed that there are two latent variables: the “Lifestyle” that we explained using smoking status, alcohol consumption, physical activity, diet, and BMI, and the “Clinical profile” that we explained using prevalent diabetes, prevalent hypercholesterolemia, and prevalent hypertension. We therefore tested the association between low education and CE, supposing an association between education and “Lifestyle”, education and “Clinical profile”, “Lifestyle” and CE, and “Clinical profile” and CE.

The good fitness of the model was tested using the root mean square error of approximation (RMSEA) and the comparative fit index (CFI), as suggested in literature [29]. SEM analysis was performed using MPLUS v.7 [Muthén LK., Muthen BO. Mplus User’s Guide. 6th ed. Los Angeles: Muthén & Muthén; 2010].

## Results

In 43,791 participants (about 472,000 person-years), with a median follow-up time of 11.26 years,we identified a total of 1,156 major CE (562 myocardial infarctions) and a total of 468 major CBVD (192 ischemic strokes and 83 hemorrhagic strokes).

Almost all the investigated variables (center, gender, age, smoking status, BMI, Menopausal status, baseline hypertension, and baseline diabetes) differed significantly between the disease-free cohort and CE and CBVD cases. Tertiles of RII were strongly different between CE cases and the disease-free cohort, but they did not differ between CBVD cases and the disease-free cohort (Table 1). All baseline exposure variables differed by RII tertiles (Table 2).

**Table 1:** Univariate analysis: baseline exposures in the disease-free cohort and in participants with cardiovascular events (CE) and cerebrovascular events (CBVD). P-values from chi square or t-test as appropriate.

	Disease-free cohort	CE	CBVD
	N (%)	N (%)	N (%)
**Center**			
*Turin*	8,768 (20.79)	333 (28.81)	90 (19.23)
*Varese*	10,905 (25.86)	385 (33.30)	207 (44.23)
*Florence*	12,132 (28.77)	163 (14.10)	92 (19.66)
*Naples*	4,720 (11.19)	102 (8.82)	32 (6.84)
*Ragusa*	5,642 (13.38)	173 (14.97)	47 (10.04)
*p-value*		<0.0001	<0.0001
**Gender**			
*Males*	12,603 (29.89)	658 (56.92)	168 (35.90)
*Females*	29,564 (70.11)	498 (43.08)	300 (64.10)
*p-value*		<0.0001	0.005
**Age (years)**			
*Mean (SD)*	49.91 (7.72)	54.19 (7.27)	55.56 (8.08)
*p-value*		<0.0001	<0.0001
**Smoking status**			
*Current*	11,200 (26.56)	418 (36.16)	137 (26.59)
*Former*	11263 (26.71)	346 (29.93)	98 (26.65)
*Never*	19,704 (46.73)	392 (33.91)	233 (46.76)
*p-value*		<0.0001	0.01
**Alcohol consumption**			
*No*	8,018 (21.49)	206 (19.69)	106 (24.37)
*Yes*	29,299 (78.51)	840 (80.31)	329 (75.63)
*p-value*		0.16	0.14
**Physical activity**			
*Low*	3,872 (10.53)	122 (11.75)	62 (14.39)
*Moderate*	21,055 (57.26)	566 (54.53)	235 (54.52)
*High*	11,842 (32.21)	350 (33.72)	134 (31.09)
*p-value*		0.18	0.03
**Italian Mediterranean Index (number of Mediterranean items)**			
*mean (SD)*	4.06 (1.83)	3.97 (1.79)	3.94 (1.78)
*p-value*		0.10	0.14
**Energy intake (kcal)**			
*means (SD)*	2,338 (725)	2,390 (803)	2,291 (774)
*p-value*		0.02	0.16
**Body mass index (kg/m2)**			
*means (SD)*	25.96 (4.06)	27.38 (3.91)	27.03 (4.48)
*p-value*		<0.0001	<0.0001
**Menopausal status**			
*Pre*	14,697 (50.25)	116 (23.29)	80 (26.67)
*Post*	14,847 (49.75)	382 (76.71)	220 (73.33)
*p-value*		<0.0001	<0.0001
**Baseline hypertension**			
*Yes*	5,365 (12.72)	267 (23.10)	125 (26.71)
*No*	36802 (87.28)	889 (76.90)	343 (73.29)
*p-value*		<0.0001	<0.0001
**Baseline hypercholesterolemia**			
*Yes*	3,536 (8.39)	148 (12.80)	47 (10.06)
*No*	38,609 (91.61)	1,008 (87.20)	420 (89.94)
*p-value*		<0.0001	0.19
**Baseline diabetes**			
*Yes*	803 (1.91)	83 (7.18)	30 (6.41)
*No*	41,342 (98.09)	1,073 (92.82)	438 (93.59)
*p-value*		<0.0001	<0.0001
**Relative Index of Inequality (RII)**			
*1*^*st*^ *tertile*	14,085 (33.40)	332 (28.72)	140 (29.91)
*2*^*nd*^ *tertile*	14,226 (33.74)	380 (32.87)	174 (37.18)
*3*^*rd*^ *tertile*	13,856 (32.86)	444 (38.41)	154 (32.91)
*p-value*		<0.0001	0.19

SD: standard deviation.

**Table 2:** Univariate analysis: baseline exposures by RII tertiles. P-values from chi square or 1-way analysis of variance, as appropriate.

	High education	Moderate education	Low education	p-value
	N (%)	N (%)	N (%)	
**Center**				
*Turin*	3,491 (23.98)	2,469 (16.71)	3,231 (22.35)	<0.0001
*Varese*	3,781 (25.86)	4,035 (27.30)	3,681 (25.47)	
*Florence*	3,749 (25.75)	4,472 (30.26)	4,166 (28.82)	
*Naples*	2,071 (14.23)	1,425 (9.64)	1,358 (9.40)	
*Ragusa*	1,465 (10.06)	2,379 (16.10)	2,018 (13.96)	
**Gender**				
*Males*	4,574 (31.42)	4,332 (29.31)	4,523 (31.29)	<0.0001
*Females*	9,983 (68.58)	10,448 (70.69)	9,931 (68.71)	
**Age (years)**				
*Mean (SD)*	50.33 (7.61)	49.48 (8.18)	50.47 (7.44)	<0.0001
**Smoking status**				
*Current*	3,835 (26.34)	4,063 (27.49)	3,857 (26.68)	<0.0001
*Former*	4,316 (29.65)	4,096 (27.71)	3,295 (22.80)	
*Never*	6,406 (44.01)	6,621 (44.80)	7,302 (50.52)	
**Alcohol consumption**				
*No*	2,203 (17.69)	2,823 (21.20)	3,304 (25.36)	<0.0001
*Yes*	10,249 (82.31)	10,495 (78.80)	9,724 (74.64)	
**Physical activity**				
*Low*	922 (8.06)	1,346 (10.29)	1,718 (13.38)	<0.0001
*Moderate*	7,070 (57.45)	7,507 (57.36)	7,279 (56.67)	
*High*	4,245 (34.49)	4,234 (32.35)	3,847 (29.95)	
**Italian Mediterranean Index (number of Mediterranean habits)**				
*Mean (SD)*	4.16 (1.86)	4.03 (1.81)	3.99 (1.79)	<0.0001
**Energy intake (kcal)**				
*Mean (SD)*	2,330 (684)	2,352 (730)	2,336 (767)	0.03
**Body mass index (kg/m**^2^)				
*Means (SD)*	25.25 (3.80)	25.82 (3.97)	26.96 (4.23)	<0.0001
**Menopausal status**				
*Pre*	4,984 (49.96)	4,816 (46.13)	5,649 (56.91)	<0.0001
*Post*	4,992 (50.04)	5,623 (53.87)	4,278 (43.09)	
**Baseline hypertension**				
*Yes*	1,800 (12.37)	1,843 (12.47)	2,114 (14.63)	<0.0001
*No*	12,757 (87.63)	12,937 (87.53)	12,340 (85.37)	
**Baseline hypercholesterolemia**				
*Yes*	1,089 (7.48)	1,315 (8.90)	1,327 (9.19)	<0.0001
*No*	13,465 (92.52)	13,455 (91.10)	13,117 (90.81)	
**Baseline diabetes**				
*Yes*	218 (1.50)	285 (1.93)	413 (2.86)	<0.0001
*No*	14,337 (98.50)	14,485 (98.07)	14,031 (97.14)	

SD: standard deviation.

An increased risk of major CE (HR = 1.39; 95% CI 1.21–1.69) was observed for the third tertile of RII with respect to the first tertile (Table 3A). This disparity was slightly reduced, but still present (HR = 1.17; 95% CI 1.01–1.37), after adjustment for lifestyle and clinical risk factors, but such reductions were due more to lifestyle than to clinical risk factors. The main lifestyle risk factors were alcohol consumption, physical activity, and BMI (S2 Table). When analyzing this association by gender, a greater effect was observed among women in the adjusted model (fully-adjusted HR = 1.36; 95% CI 1.04–1.79), while the association in men disappeared (fully-adjusted HR = 1.08; 95% 0.89–1.31). A similar pattern was found when analyzing participants with myocardial infarction only (S1 Table, Panel A).

**Table 3:** Major cardiovascular events (Panel A) and major cerebrovascular events (Panel B): crude and adjusted Cox models. Model 1 is adjusted by smoking status, alcohol consumption, physical activity, Italian Mediterranean Index, energy intake, and body mass index. Model 2 is adjusted by baseline hypertension, baseline hypercholesterolemia, and prevalent diabetes. Model 3 is adjusted as in Model 1+ Model 2. All models are adjusted by age and sex and stratified by center.

Panel A) Major coronary events (M = 658; F = 498)									
		Crude HR	95% CI	Adj 1 HR	95% CI	Adj 2 HR	95% CI	Adj 3 HR	95% CI
Men and women	High education	Ref		Ref		Ref		Ref	
	Moderate educ	1.2	1.04–1.39	1.14	0.98–1.34	1.19	1.03–1.38	1.15	0.98–1.34
	Low education	1.39	1.21–1.61	1.19	1.02–1.38	1.35	1.17–1.56	1.17	1.01–1.37
	p for trend	<0.0001		0.03		<0.0001		0.04	
Men	High education	Ref		Ref		Ref		Ref	
	Moderate educ	1.13	0.93–1.38	1.04	0.86–1.27	1.13	0.93–1.38	1.04	0.86–1.27
	Low education	1.23	1.02–1.49	1.08	0.90–1.30	1.22	1.01–1.47	1.08	0.89–1.31
	p for trend	0.03		0.45		0.04		0.44	
Women	High education	Ref		Ref		Ref		Ref	
	Moderate educ	1.32	1.05–1.66	1.34	1.04–1.74	1.3	1.03–1.63	1.35	1.04–1.74
	Low education	1.71	1.37–2.14	1.4	1.07–1.83	1.6	1.28–2.01	1.36	1.04–1.79
	p for trend	<0.0001		0.01		<0.0001		0.01	
Panel B) Major cerebrovascular events (M = 168; F = 300)									
		Crude HR	95% CI	Adj 1 HR	95% CI	Adj 2 HR	95% CI	Adj 3 HR	95% CI
Men and women	High education	Ref		Ref		Ref		Ref	
	Moderate educ	1.24	1.00–1.56	1.2	0.95–1.52	1.22	0.98–1.54	1.2	0.95–1.52
	Low education	1.17	0.93–1.47	1.11	0.87–1.42	1.11	0.88–1.40	1.08	0.85–1.38
	p for trend	0.19		0.41		0.38		0.54	
Men	High education	Ref		Ref		Ref		Ref	
	Moderate educ	1.01	0.69–1.48	0.94	0.64–1.39	1.01	0.69–1.48	0.95	0.64–1.40
	Low education	1.18	0.81–1.70	1.1	0.76–1.60	1.17	0.81–1.69	1.11	0.76–1.61
	p for trend	0.38		0.6		0.39		0.58	
Women	High education	Ref		Ref		Ref		Ref	
	Moderate educ	1.38	1.04–1.82	1.38	1.02–1.87	1.35	1.02–1.78	1.38	1.02–1.87
	Low education	1.17	0.87–1.57	1.13	0.81–1.57	1.09	0.81–1.47	1.08	0.78–1.51
	p for trend	0.29		0.48		0.57		0.64	

HR: hazard ratio, CI: confidence interval, Adj: adjusted.

No significant association was found between SES and CBVD when males and females were analyzed together, although an increased risk of CBVD was seen for the second tertile of RII with respect to the first in women (fully-adjusted HR = 1.38; 95% CI 1.02–1.87) without any suggestion of confounding (Table 3B). Subtype analyses showed a similar pattern, but it was not statistically significant, probably due to the small sample sizes (S1 Table, Panels B and C).

The model proposed for SEM analysis (Fig 1) gave a satisfactory fit to data (RMSEA estimate = 0.028, and CFI = 0.95) and the results showed that, after multiple adjustments, the relationship between low education and CE was still present (p-value = 0.01) as well as the relationship between “lifestyle” and CE (p-value = 0.005), while the relationship between “clinical profile” and CE disappeared (p-value = 0.54)

We observed a strong relationship between RII and diabetes (adjusted HR = 1.69; 95% CI 1.41–2.04), while a smaller or no association was found for hypercholesterolemia (adjusted HR = 1.13; 95% CI 1.03–1.25) and hypertension (adjusted HR = 1.05; 95% CI 0.97–1.13) (Table 4). The same behavior was observed when performing gender-separated analyses, with higher risks for women (data not shown).

**Table 4:** Logistic regression models for the association between the relative index of inequality (RII) and clinical risk factors: hypertension at baseline (Panel A), hypercholesterolemia at baseline (Panel B), and diabetes at baseline (Panel C). Multivariate models are adjusted by age, sex, center, smoking status, physical activity, alcohol consumption, Dietary Mediterranean index, and energy intake.

Panel A) Prevalent hypertension (M = 1,722; F = 4,035)				
	Crude HR	95% CI	Adj 1 HR	95% CI
High education	Ref		Ref	
Moderate educ	1.01	0.94–1.08	0.96	0.89–1.04
Low education	1.21	1.14–1.30	1.05	0.97–1.13
p for trend	<0.0001		0.21	
Panel B) Prevalent hypercholesterolemia (M = 1,268; F = 2,463)				
	Crude HR	95% CI	Adj 1 HR	95% CI
High education	Ref		Ref	
Moderate educ	1.21	1.11–1.31	1.08	0.98–1.18
Low education	1.25	1.15–1.36	1.13	1.03–1.25
p for trend	<0.0001		0.01	
Panel C) Prevalent diabetes (M = 298; F = 618)				
	Crude HR	95% CI	Adj 1 HR	95% CI
High education	Ref		Ref	
Moderate educ	1.29	1.08–1.55	1.24	1.02–1.51
Low education	1.94	1.64–2.28	1.69	1.41–2.04
p for trend	<0.0001		<0.0001	

HR: hazard ratio, CI: confidence interval, Adj: adjusted.

## Discussion

The results from the EPICOR study provide evidence of the effect of the educational component of SES on the incidence of CE in recent years in Italy, while no such effect was seen for CBVD (even if a slight increase of risk was found for women with moderate education respect to women with high education). SES is a common concept in social and medical research, but it can be measured with different and not always interchangeable indicators [14]. In this study we measured SES with educational level and it is shown that education is not affected by poor health in adulthood and reflects childhood and adolescent SES based on the SES of parents. At the same time it partially reflects adult income, and social prestige, also in unemployed women. To overcome the issue of unbalance in the gender and age distribution of participants across educational levels we calculated the RII, that expresses inequality within the socioeconomic continuum.

A recent meta-analysis [30] included 70 case-control or cohort studies and showed a significant increase in the risk of myocardial infarction in people with the lowest SES socioeconomic categories, regardless of the SES indicator used (RR = 1.34; 95% CI 1.22–1.47 for the lowest educational group). In another quantitative meta-analysis [13], Baldi et al observed that at least a difference of 10 years of education is needed to obtain a protective effect on CE incidence and mortality.

The association between RII and major CE in our study was decisively attenuated by the adjustment for lifestyle risk factors, though it remained statistically significant and maintained a dose-response gradient. The lifestyle variables significantly associated to CE were BMI, alcohol consumption, and physical activity. Smoking status was more common in men with a low educational level, while it shows an inverse pattern in women. This relationship between smoking and CE could support the existing evidence that smoking is more common among women with a high level of education in Southern Europe [31]. In previous studies on this topic, the HR for CE i significantly decreased after adjustment for lifestyle factors (mainly alcohol consumption, smoking, obesity, and physical activity) [32–36], but the majority of them looked at the relationship between SES and CE mortality, which may explain the different roles of risk factors in explaining the association, particularly for smoking.

In contrast to the important role of lifestyle risk factors, the association between RII and major CE in our study was only slightly attenuated by adjustment for clinical risk factors, with diabetes explaining largest proportion. This result is coherent with some [37], but not all previous studies [33, 38]. Blood pressure played a moderate role in explaining different CE incidence with respect to SES, which was also shown in a study on Scottish men and women [33].

Some studies have assessed the role of lifestyle and clinical risk factors in the same analysis, but very few of them focused on the incidence of CE instead of mortality [34, 39–41]. A general study on the incidence and mortality of CE found that adjustment for lifestyle risk factors explained a variable proportion of the relative education-related inequality in CE incidence and mortality, but a residual association persisted. Several studies from the UK [16, 18, 42] have reported results similar to ours, showing that adjustment for smoking and other lifestyle risk factors reduced the excess risk of CE associated with low SES more than any clinical risk factor.

As in other cohort studies that used education as an indicator of SES [43–45], in the EPICOR study we observed an excess of risk for women in the lowest tertile of RII with respect to men. However, previous studies showed that classical clinical risk factors such as diabetes and hypertension seem to be more strongly related to CE in women and more associated with SES [32, 43], whereas adjusting for lifestyle risk factors in our study attenuated the risk in women more evidently than adjusting for clinical risk factors. Our results indicated that the educational gradient in BMI, physical activity, and alcohol consumption may account for a large proportion of the differences among men and women, in agreement with a US survey [45].

In our study, results from Cox models were strengthened by SEM analysis; in fact, it emerged that “lifestyle” characteristics (smoking status, physical activity, diet, BMI, and alcohol consumption) showed a greater impact than “clinical profile” characteristics (prevalent diabetes, hypercholesterolemia, hypertension) on explaining the excess of CE incidence among less educated subjects.

We did not observe any statistically significant association between CBVD and education in men or women. In women, only the second tertile versus the first tertile of RII showed an increased risk for stroke, which was attenuated after adjusting for clinical risk factors. We do not have a clear explanation for the increase in risk we observed in the middle RII tertile, while our evidence of a higher risk of stroke among women than men was coherent with previous studies [46–47].

The difference in results our with respect to CBVD, even though lifestyle and clinical risk factors for ischemic stroke were similar, may be due in part to the differential etiological role played by blood pressure level in CE and CBVD. Indeed, blood pressure was more closely associated with CE in our study, and we did not find a clear association between hypertension and RII.

Another important aim of the EPICOR study was to analyze the prevalence the major clinical risk factors hypertension, hypercholesterolemia and type 2 diabetes and determine their association with education. Few studies on the association between SES and CE/CBVD have analyzed clinical risk factors separately; therefore, it is not clear which risk factors may be influential in explaining socioeconomic inequality in diseases [18, 38, 48, 49]. The oldest studies showed a modest or no association between hypertension and hypercholesterolemia and SES [48], while a clear association was found in more recent publications [18, 38, 49]. The results of the EPICOR study showed no association with hypertension and a modest association with hypercholesterolemia (adjusted HR = 1.05; 95% CI 0.97–1.13 and adjusted HR = 1.13; 95% CI 1.03–1.25, respectively). As previously reported, the EPICOR study showed an important association between diabetes and RII (adjusted HR = 1.69; 95% CI 1.41–2.04) [3, 50].

Our study exhibits strengths and limitations. We analyzed incident cases of CE and CBVD from a cohort study. This allowed us to disaggregate the effect of education on disease occurrence from the differential access to adequate care and/or the delay in hospital admission for emergencies. Furthermore, this is one of the few cohort studies in which the cardiovascular disease status has been validated by a cardiologist using original medical notes. We also used information about potential risk factors and were able to build different multivariate models to identify the intermediate variables that better explained the observed inequalities.

This study also has some limitations. First, the population under study is entirely represented by white men and women belonging mainly to medium social classes. This could imply a lower range of exposure and an underestimate of the real social inequalities in disease incidence. Second, we measured SES using education and not income. Usually, income is chosen as the nearest proxy for SES, but it has been shown that, as a matter of fact, it is not a good proxy for wealth [51]; moreover, income is a sensitive item and the refusal rate when this information is collected is high, especially in Europe [52]. Third, risk factors were measured at baseline only (during the 90ies); anyway, this assures the respect of the temporality assumption for causation [53] Furthermore, residual confounding by unmeasured variables, such as psychological factors and stress, likely occurred in the study.

In conclusion, our results suggest that the observed differences in CE risk with respect to educational level may result from differential exposure to lifestyle risk factors (in particular BMI, physical activity, and smoking in males) more than to clinical risk factors (among which the major role is played by diabetes).

## Supporting Information

S1 Table Myocardial infarction (Panel A), Ischemic Stroke (Panel B) and Hemorhagic Stroke (Panel C): crude and adjusted Cox models. Model 1 is adjusted by smoking status, alcohol consumption, physical activity, Italian Mediterranean Index, energy intake, and body mass index. Model 2 is adjusted by baseline hypertension, baseline hypercholesterolemia, and prevalent diabetes. Model 3 is adjusted as in Model 1+ Model 2. All models are adjusted by age and sex and stratified by center.
(PDF)

S2 Table Association between Relative Index of Inequality and Major coronary events. Models with addition of variables.
(PDF)

S3 Table Association between Relative Index of Inequality and Major cerebrovascular events. Models with addition of variables.
(PDF)
