# Supplementary material for: The Association between Educational Level and Cardiovascular and Cerebrovascular Diseases within the EPICOR Study: New Evidence for an Old Inequality Problem
Source: PLoS One. 2016 Oct 6;11(10):e0164130. doi: 10.1371/journal.pone.0164130 (PMC5053474; doi:10.1371/journal.pone.0164130)
Supplement: S1 Table — Model 1 is adjusted by smoking status, alcohol consumption, physical activity, Italian Mediterranean Index, energy intake, and body mass index. Model 2 is adjusted by baseline hypertension, baseline hypercholesterolemia, and prevalent diabetes. Model 3 is adjusted as in Model 1+ Model 2. All models are adjusted by age and sex and stratified by center. (PDF) [file pone.0164130.s001.pdf]

S1 Table. Myocardial infarction (Panel A), Ischemic Stroke (Panel B) and Hemorhagic Stroke (Panel C): crude and adjusted Cox models. Model 1 is adjusted by smoking status, alcohol consumption, physical activity, Italian Mediterranean Index, energy intake, and body mass index. Model 2 is adjusted by baseline hypertension, baseline hypercholesterolemia, and prevalent diabetes. Model 3 is adjusted as in Model 1+ Model 2. All models are adjusted by age and sex and stratified by center.

Panel A) Myocardial Infarction (M=367; F=195)									
		Crude HR	95% CI	Adj 1 HR	95 % CI	Adj 2 HR	95% CI	Adj 3 HR	95% CI
Men and women	1st tertile	Ref		Ref		Ref		Ref	
	2nd tertile	1.15	0.93-1.42	1.07	0.85-1.33	1.14	0.92-1.41	1.06	0.85-1.33
	3rd tertile	1.36	1.11-1.67	1.10	0.89-1.37	1.33	1.09-1.63	1.10	0.88-1.36
	p for trend	0.003		0.39		0.005		0.41	
Men	1st tertile	Ref		Ref		Ref		Ref	
	2nd tertile	1.06	0.82-1.38	0.99	0.76-1.29	1.06	0.82-1.38	0.98	0.75-1.28
	3rd tertile	1.21	0.95-1.55	1.05	0.82-1.36	1.20	0.94-1.54	1.06	0.82-1.36
	p for trend	0.12		0.68		0.14		0.66	
Women	1st tertile	Ref		Ref		Ref		Ref	
	2nd tertile	1.35	0.94-1.96	1.35	0.89-2.04	1.33	0.92-1.92	1.35	0.89-2.05
	3rd tertile	1.76	1.23-2.51	1.33	0.86-2.06	1.65	1.15-2.35	1.29	0.84-2.00
	p for trend	0.002		0.20		0.006		0.26	
Panel B) Ischemic Stroke (M=86; F=106)									
		Crude HR	95% CI	Adj 1 HR	95 % CI	Adj 2 HR	95% CI	Adj 3 HR	95% CI
Men and women	1st tertile	Ref		Ref		Ref		Ref	
	2nd tertile	1.25	0.87-1.78	1.23	0.85-1.81	1.23	0.86-1.76	1.24	0.85-1.82
	3rd tertile	1.31	0.92-1.87	1.32	0.90-1.93	1.22	0.86-1.75	1.26	0.86-1.85
	p for trend	0.13		0.16		0.27		0.24	
Men	1st tertile	Ref		Ref		Ref		Ref	
	2nd tertile	1.01	0.57-1.80	0.93	0.52-1.66	1.01	0.57-1.79	0.94	0.52-1.68
	3rd tertile	1.65	1.00-2.75	1.48	0.88-2.49	1.63	0.98-2.71	1.49	0.89-2.50
	p for trend	0.04		0.11		0.05		0.11	
Women	1st tertile	Ref		Ref		Ref		Ref	
	2nd tertile	1.43	0.90-2.26	1.54	0.92-2.59	1.40	0.88-2.21	1.57	0.93-2.63
	3rd tertile	1.08	0.65-1.79	1.14	0.64-2.04	0.97	0.59-1.61	1.06	0.59-1.91
	p for trend	0.74		0.66		0.93		0.84	
Panel C) Hemorrhagic Stroke (M=26; F=57)									
		Crude HR	95% CI	Adj 1 HR	95 % CI	Adj 2 HR	95% CI	Adj 3 HR	95% CI
Men and women	1st tertile	Ref		Ref		Ref		Ref	
	2nd tertile	1.17	0.70-1.96	0.97	0.56-1.65	1.16	0.69-1.95	0.97	0.56-1.66
	3rd tertile	0.97	0.56-1.67	0.79	0.45-1.41	0.94	0.54-1.62	0.78	0.44-1.39
	p for trend	0.91		0.43		0.83		0.41	
Men	1st tertile	Ref		Ref		Ref		Ref	
	2nd tertile	0.61	0.22-1.67	0.50	0.17-1.48	0.61	0.22-1.68	0.51	0.17-1.51
	3rd tertile	1.03	0.43-2.47	1.00	0.41-2.43	1.04	0.43-2.51	1.04	0.43-2.55
	p for trend	0.96		0.99		0.93		0.93	
Women	1st tertile	Ref		Ref		Ref		Ref	
	2nd tertile	1.47	0.79-2.73	1.28	0.68-2.43	1.46	0.78-2.71	1.28	0.67-2.43
	3rd tertile	0.91	0.45-1.83	0.74	0.34-1.58	0.88	0.44-1.77	0.73	0.34-1.57
	p for trend	0.83		0.49		0.75		0.47	
