# Supplementary material for: The Association between Educational Level and Cardiovascular and Cerebrovascular Diseases within the EPICOR Study: New Evidence for an Old Inequality Problem
Source: PLoS One. 2016 Oct 6;11(10):e0164130. doi: 10.1371/journal.pone.0164130 (PMC5053474; doi:10.1371/journal.pone.0164130)
Supplement: S2 Table — Models with addition of variables. (PDF) [file pone.0164130.s002.pdf]

S2 Table. Association between Relative Index of Inequality and Major coronary events. Models with addition of variables

		Male and Female		Male		Female	
Major coronary events		HR	95% CI	HR	95 % CI	HR	95% CI
Crude model	1st tertile	Ref		Ref		Ref	
	2nd tertile	1.20	1.03-1.39	1.13	0.93-1.38	1.32	1.05-1.66
	3rd tertile	1.39	1.21-1.61	1.23	1.02-1.49	1.71	1.37-2.14
	p for trend	<0.0001		0.03		<0.0001	
Smoking adjustment	1st tertile	Ref		Ref		Ref	
	2nd tertile	1.19	1.02-1.38	1.08	0.89-1.31	1.36	1.08-1.71
	3rd tertile	1.38	1.20-1..59	1.15	0.96-1.39	1.81	1.45-2.27
	p for trend	<0.0001		0.13		<0.0001	
Alchool adjustment	1st tertile	Ref		Ref		Ref	
	2nd tertile	1.21	1.04-1.41	1.14	0.93-1.39	1.35	1.05-1.74
	3rd tertile	1.30	1.11-1.51	1.23	1.02-1.48	1.51	1.17-1.96
	p for trend	0.001		0.03		0.002	
Physical activity adjustment	1st tertile	Ref		Ref		Ref	
	2nd tertile	1.20	1.03-1.40	1.11	0.91-1.35	1.37	1.06-1.77
	3rd tertile	1.30	1.11-1.51	1.22	1.01-1.48	1.51	1.16-1.96
	p for trend	0.001		0.04		0.002	
Nutrition adjustment	1st tertile	Ref		Ref		Ref	
	2nd tertile	1.20	1.04-1.39	1.13	0.93-1.38	1.32	1.05-1.66
	3rd tertile	1.39	1.21-1.61	1.24	1.02-1.49	1.71	1.37-2.13
	p for trend	<0.0001		0.03		<0.0001	
BMI adjustment	1st tertile	Ref		Ref		Ref	
	2nd tertile	1.17	1.00-1.35	1.10	0.91-1.34	1.28	1.02-1.61
	3rd tertile	1.30	1.13-1.51	1.17	0.97-1.41	1.59	1.27-1.99
	p for trend	<0.0001		0.11		<0.0001	
Menopausal status adjustment	1st tertile	-		-		Ref	
	2nd tertile					1.32	1.05-1.67
	3rd tertile					1.66	1.32-2.07
	p for trend					<0.0001	
Diabetes adjustment	1st tertile	Ref		Ref		Ref	
	2nd tertile	1.20	1.03-1.39	1.13	0.93-1.37	1.32	1.04-1.65
	3rd tertile	1.36	1.18-1.57	1.21	1.01-1.46	1.65	1.32-2.07
	p for trend	<0.0001		0.04		<0.0001	
Hypercholesterolemia adjustment	1st tertile	Ref		Ref		Ref	
	2nd tertile	1.20	1.03-1.39	1.13	0.93-1.37	1.32	1.05-1.66
	3rd tertile	1.37	1.19-1.58	1.22	1.01-1.47	1.68	1.34-2.10
	p for trend	<0.0001		0.04		<0.0001	
Hypertension adjustment	1st tertile	Ref		Ref		Ref	
	2nd tertile	1.20	1.03-1.39	1.13	0.93-1.38	1.31	1.04-1.65
	3rd tertile	1.38	1.20-1.59	1.24	1.03-1.50	1.66	1.33-2.08
	p for trend	<0.0001		0.02		<0.0001	
