# Supplementary material for: The Association between Educational Level and Cardiovascular and Cerebrovascular Diseases within the EPICOR Study: New Evidence for an Old Inequality Problem
Source: PLoS One. 2016 Oct 6;11(10):e0164130. doi: 10.1371/journal.pone.0164130 (PMC5053474; doi:10.1371/journal.pone.0164130)
Supplement: S3 Table — Models with addition of variables. (PDF) [file pone.0164130.s003.pdf]

**S3 Table. Association between Relative Index of Inequality and Major cerebrovascular events. Models with addition of variables**

| Major cerebrovascular events | | Male and Female | | Male | | Female | |
|---|---|---|---|---|---|---|---|
| | | HR | 95% CI | HR | 95 % CI | HR | 95% CI |
| Crude model | 1st tertile | Ref | | Ref | | Ref | |
| | 2nd tertile | 1.24 | 1.00-1.56 | 1.01 | 0.69-1.48 | 1.38 | 1.04-1.82 |
| | 3rd tertile | 1.17 | 0.93-1.47 | 1.18 | 0.82-1.70 | 1.17 | 0.87-1.57 |
| | p for trend | 0.19 | | 0.38 | | 0.29 | |
| Smoking adjustment | 1st tertile | Ref | | Ref | | Ref | |
| | 2nd tertile | 1.24 | 0.99-1.55 | 0.98 | 0.67-1.45 | 1.38 | 1.04-1.82 |
| | 3rd tertile | 1.15 | 0.91-1.45 | 1.12 | 0.78-1.62 | 1.17 | 0.87-1.58 |
| | p for trend | 0.24 | | 0.53 | | 0.30 | |
| Alchool adjustment | 1st tertile | Ref | | Ref | | Ref | |
| | 2nd tertile | 1.23 | 0.97-1.55 | 0.99 | 0.67-1.45 | 1.38 | 1.03-1.86 |
| | 3rd tertile | 1.17 | 0.92-1.49 | 1.18 | 0.82-1.71 | 1.18 | 0.85-1.62 |
| | p for trend | 0.20 | | 0.36 | | 0.32 | |
| Physical activity adjustment | 1st tertile | Ref | | Ref | | Ref | |
| | 2nd tertile | 1.25 | 0.99-1.58 | 1.00 | 0.68-1.47 | 1.42 | 1.05-1.92 |
| | 3rd tertile | 1.19 | 0.93-1.51 | 1.17 | 0.81-1.69 | 1.23 | 0.89-1.69 |
| | p for trend | 0.17 | | 0.39 | | 0.21 | |
| Nutrition adjustment | 1st tertile | Ref | | Ref | | Ref | |
| | 2nd tertile | 1.24 | 1.00-1.55 | 1.00 | 0.68-1.47 | 1.38 | 1.04-1.81 |
| | 3rd tertile | 1.16 | 0.92-1.47 | 1.16 | 0.81-1.68 | 1.17 | 0.87-1.57 |
| | p for trend | 0.2 | | 0.41 | | 0.29 | |
| BMI adjustment | 1st tertile | Ref | | Ref | | Ref | |
| | 2nd tertile | 1.22 | 0.98-1.53 | 1.00 | 0.68-1.47 | 1.37 | 1.02-1.77 |
| | 3rd tertile | 1.12 | 0.88-1.41 | 1.16 | 0.80-1.68 | 1.09 | 0.81-1.48 |
| | p for trend | 0.36 | | 0.42 | | 0.56 | |
| Menopausal status adjustment | 1st tertile | - | | - | | Ref | |
| | 2nd tertile | | | | | 1.38 | 1.04-1.82 |
| | 3rd tertile | | | | | 1.16 | 0.86-1.56 |
| | p for trend | | | | | 0.33 | |
| Diabetes adjustment | 1st tertile | Ref | | Ref | | Ref | |
| | 2nd tertile | 1.24 | 0.99-1.55 | 1.01 | 0.69-1.48 | 1.37 | 1.04-1.81 |
| | 3rd tertile | 1.14 | 0.91-1.44 | 1.17 | 0.81-1.69 | 1.14 | 0.85-1.53 |
| | p for trend | 0.26 | | 0.41 | | 0.39 | |
| Hypercholesterolemia adjustment | 1st tertile | Ref | | Ref | | Ref | |
| | 2nd tertile | 1.24 | 1.00-1.55 | 1.01 | 0.69-1.48 | 1.37 | 1.04-1.81 |
| | 3rd tertile | 1.15 | 0.92-1.45 | 1.18 | 0.82-1.71 | 1.14 | 0.85-1.54 |
| | p for trend | 0.22 | | 0.37 | | 0.37 | |
| Hypertension adjustment | 1st tertile | Ref | | Ref | | Ref | |
| | 2nd tertile | 1.23 | 0.98-1.54 | 1.01 | 0.69-1.48 | 1.36 | 1.03-1.79 |
| | 3rd tertile | 1.14 | 0.91-1.43 | 1.18 | 0.82-1.71 | 1.13 | 0.84-1.52 |
| | p for trend | 0.27 | | 0.37 | | 0.42 | |
